# Health care costs associated with hospital acquired complications in patients with chronic kidney disease

**DOI:** 10.1186/s12882-017-0784-2

**Published:** 2017-12-28

**Authors:** Babak Bohlouli, Terri Jackson, Marcello Tonelli, Brenda Hemmelgarn, Scott Klarenbach

**Affiliations:** 1 Department of Medicine, University of Alberta, 11-112R Clinical Sciences Building, 8440-112 Street, Edmonton, AB T6G2G3 Canada; 2 University of Melbourne, Northern Clinical Research Centre, The Northern Hospital, 185 Cooper St, Epping, VIC 3076 Australia; 3 Department of Medicine, Cumming School of Medicine, University of Calgary, 7th Floor, TRW Building, 3280 Hospital Drive NW, Calgary, AB T2N 4Z6 Canada; 4 Department of Medicine, Cumming School of Medicine, University of Calgary, 3D10, TRW Building 3280 Hospital Drive NW, Calgary, AB Canada

**Keywords:** Chronic kidney disease, Healthcare costs, Hospital acquired complication, Readmission

## Abstract

**Background:**

Patients with CKD are at increased risk of potentially preventable hospital acquired complications (HACs). Understanding the economic consequences of preventable HACs, may define the scope and investment of initiatives aimed at prevention.

**Methods:**

Adult patients hospitalized from April, 2003 to March, 2008 in Alberta, Canada comprised the study cohort. Healthcare costs were determined and categorized into ‘index hospitalization’ including hospital cost and in-hospital physician claims, and ‘post discharge’ including ambulatory care cost, physician claims, and readmission costs from discharge to 90 days. Multivariable regression was used to estimate the incremental healthcare costs associated with potentially preventable HACs.

**Results:**

In fully adjusted models, the median incremental index hospitalization cost was CAN-$6169 (95% CI; 6003–6336) in CKD patients with ≥1 potentially preventable HACs, compared with those without. Post-discharge incremental costs were 1471(95% CI; 844–2099) in those patients with CKD who developed potentially preventable HACs within 90 days after discharge compared with patients without potentially preventable HACs. Additionally, the incremental costs associated with ≥1 potentially preventable HACs within 90 days from admission in patients with CKD were $7522 (95% CI; 7219–7824). A graded relation of the incremental costs was noted with the increasing number of complications. In patients without CKD but with ≥1 preventable HACs incremental costs within 90 days from hospital admission was $6688 (95% CI: 6612–6723).

**Conclusions:**

Potentially preventable HACs are associated with substantial increases in healthcare costs in people with CKD. Investment in implementing targeted strategies to reduce HACs may have a significant benefit for patient and health system outcomes.

## Background

With escalating costs of medical care and focus on healthcare system sustainability, increasing attention is being placed on gaining efficiency and maximizing value of healthcare. Hospital acquired complication (HACs) are defined as unintended clinical conditions, distinct from the admitting diagnosis, that may occur in hospitalized patients. HACs are common and occur in 2.9 to 23% of hospitalizations [1–4]. They are associated with poor outcomes including higher mortality, greater 30-day readmission, longer length of stay in hospital, and incremental costs compared with those without complications in general hospitalized patient populations [5, 6]. In the general hospitalized patient population, HACs are associated with an additional CAN $10,866 per patient, or more than double the mean cost of an uncomplicated hospital admission, and estimated to add 17.3% to treatment costs [4]. Complications deemed to be potentially preventable result in incremental healthcare costs [7, 8]. Patients with chronic kidney disease (CKD) are hospitalized frequently [9] and data from the Canada and the US have demonstrated that CKD patients are at higher risk of developing potentially preventable HACs compared with patients without CKD [10, 11].

To our knowledge, the economic consequences of HACs, including those complications that are potentially preventable, have not been determined in patients with CKD. The incremental healthcare costs associated with potentially preventable HACs is important to frame the potential benefit of strategies aimed at reducing complications. Understanding the costs associated with potentially preventable complications may inform prioritization of prevention efforts in patients with CKD and may inform the scope of investment in prevention efforts.

## Methods

### Design, setting, study population and characteristics

**Fig. 1:**
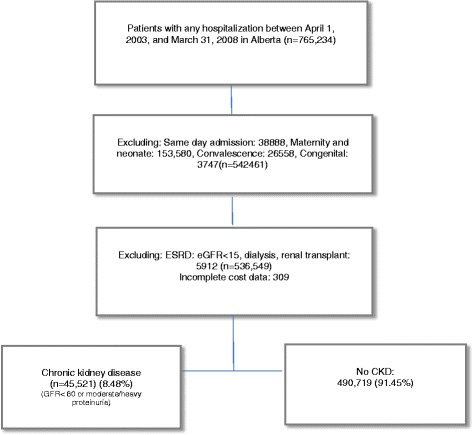
Study flowchart to construct cohort of patients with CKD

We assembled a cohort of hospitalized patients as previously described [10]. Briefly, all adults (age ≥ 18) in Alberta hospitalized from April 1, 2003 to March 31, 2008 (Fig. 1) were included, and the first hospitalization (excluding maternity/neonatal, congenital malformation, convalescence, same day admission) was defined as the index encounter. CKD and its severity, comorbid conditions, all HACs, and potentially preventable HACs were determined using Alberta Kidney Disease Network (AKDN). The primary exposure variable of CKD was defined by eGFR <60 ml/min/1.73m^2^and /or moderate to high proteinuria defined as an albumin/creatinine ratio > 3–30 mg/mmol or protein/creatinine ratio > 15–50 mg/mmol or dipstick with ≥2 proteinuria in the year prior to index hospitalization. All outpatient eGFR measurements in the time frame from 365 days to 90 days prior to admission were considered; we excluded eGFR measurement within 3 months of admission to ensure that acute kidney injury (AKI) did not impact CKD determination [12]. Patients with end stage renal disease (ESRD) and dialyses were excluded from cohort. List of the comorbid conditions was included 16 diseases; cancer, cerebrovascular disease, congestive heart failure, COPD, dementia, diabetes with complications and without complications, HIV/AIDS, metastatic solid tumor, myocardial infarction, mild liver disease, moderate/severe liver disease, para/hemiplegia, peptic ulcer disease, peripheral vascular diseases, rheumatologic disease [13]. In Canada, hospital administrative data includes “diagnoses type 2” which identifies all new diagnosis or complications that occur during hospitalization (not admitting or pre-existing diagnoses). In the US, 3 M health system released list of hospital complications deemed to be potentially preventable. Briefly, panels of clinicians (two general internists and one pediatrician supplemented by surgical, obstetric specialist as needed) reviewed each of approximately 14,400 diagnosis values in the ICD-9-CM coding scheme and classified 1562 diagnostic codes as potentially preventable in-hospital complications [8, 14]. Pulmonary embolism, deep vein thrombosis, major gastro-intestinal complications with significant bleeding, and decubitus ulcer are examples of potentially preventable complications. That information was used to identify 63 potentially preventable complications by manually re-mapping ICD 9 diagnostic codes to ICD 10 CA. (Appendix A).

Costs of inpatient care were determined using the Canadian Institute for Health Information methods [15], developed to estimate average costs of services delivered to patients in all acute care facilities. Each case is assigned to one of 17 major clinical categories and case mix group (CMGs) according to clinical and similarities in health resource use, modified by complexity. Furthermore, The National Ambulatory Care Reporting System was used to analyze outpatients’ costs. This data set was developed by Canadian Institute for Health Information and includes data for all hospital-based and community-based ambulatory care including: day surgery, outpatient and community-based clinics, emergency department visits, ambulatory interventions, rehabilitation and clinic visits except for telephone visits and direct diagnostic imaging. Outpatient pharmaceutical costs data set was not available and was not included. More details on costing are provided in Appendix B. Most physicians in Alberta are paid for each service they deliver through fee-for-service where compensation occurs with submission of a claim.

We considered healthcare cost during three distinct but overlapping intervals. The first interval was the index hospitalization including costs of hospitalization and physician claims during hospitalization. The second Interval was from discharge date to 90 days and included ambulatory care costs, hospitalization costs of readmission, and physician claims. The third interval began from hospital admission and ended at 90 days following admission: hospitalizations lasting longer than 90 days (*n* = 371) were excluded. In the post-discharge period, only the first readmission was included (if it occurred); the costs of readmissions that extended beyond the 90 day observation period were included in the main analyses, but excluded in sensitivity analysis (*n* = 508). We have reported all costs in 2008 $CA.

### Statistical analysis

The primary analysis was conducted in a cohort of patients with CKD comparing incremental costs in patients with potentially preventable HACs to those without; a second analysis considered subjects with and without CKD. Median, mean, and standard deviation of each cost category was determined. Data transformation, e.g. logarithmic, is frequently used with skewed cost data, although may not be required with large sample sizes [16] and in this study data transformation was not performed. All primary analyses used regression models. To analyze incremental costs associated with ≥1 potentially preventable HACs multivariable regression modeling was used. Population attributable risk percentages estimates are useful for providing a measure of the proportion of outcomes that can be attributed to individual or multiple causal factors Poisson regression was used to calculate population attributable risk and this formula was used:

“PAR%= Pe (RRe-1) / [1 + Pe (RRe-1)]”, where *“RRe” represents relative risk of outcomes due to HACs and “Pe” represents proportion of outcomes in cohort of patients with and without HACs* [17].

Additional regression models were conducted to assess the sensitivity of outcomes with ≥1 potentially preventable HAC within quantiles of LOS which may allow one to assess how any quantile of a conditional distribution changes with patient characteristics, for example LOS (0–25%, 26–50%). The fully adjusted models included reason for admission, age, gender, admission type (categorical; urgent vs. elective defined in hospital administrative data), length of stay (LOS), severity of CKD (where appropriate), and 16 co-morbid conditions. All analyses were also adjusted for HACs deemed not to be preventable. In additional analyses, we categorized the number of potentially preventable HACs as one, 2–3, 4–5, and > 5. We stratified the cohort of subjects with CKD to examine moderate risk, high risk, and very high risk CKD as defined by Kidney Disease, Improving Global Outcomes [18]. In sensitivity analyses, we considered all HACs (both preventable and non-preventable) as the exposure variable. LOS is a main driver of hospital cost, and as such we assessed the association of cost with potentially preventable HACs in patients grouped by LOS categories using a quantile model. The analysis was undertaken using Stata, version 13. The Health Research Ethics Board of the University of Alberta and University of Calgary approved the study.

## Results

### Patients’ characteristics

Baseline characteristics have been previously described [10] and are presented in Table 1. The unadjusted median length of hospital stay in patients without potentially preventable HAC was 5 days (25th - 75th percentile; 2–9 days) compared with 13 days (25th - 75th percentile; 7–29 days) in patients with potentially preventable HACs (Table 1).

**Table 1:** Characteristics of patients

			All patients	With preventable HAC	No preventable HAC
Demographics					
Number of subjects (%)		With CKD	45,521 (100)	4463 (9.8)	41,058 (92.2)
		No CKD	490,719 (100)	26,374 (5.4)	464,442 (94.6)
Age, mean		With CKD	72	75	72
		No CKD	50	61	50
Male (%)		With CKD	43	46.02	43.4
		No CKD	50	51	50
Top 3 most responsible diagnosis (reason for admission) categories (%) in patients with CKD					
Disease of circulatory system (%)		With CKD	21	29	20
		No CKD	12	23	11
Neoplasm (%)		With CKD	11	15	11
		No CKD	9	17	9
Disease of the digestive system (%)		With CKD	11.1	10	11.3
		No CKD	14	13	14
Admission type (Urgent %)		With CKD	71	67	72
		No CKD	71	65	70
Preventable HACs (n) (%)	Zero	With CKD	41,058 (92)	–	41,058 (92)
		No CKD	464,442 (94)		464,442 (94)
	One	With CKD	–	2836 (6.8)	–
		No CKD		18,083 (3.8)	
	2–3	With CKD	–	1225 (3)	–
		No CKD		6488 (1.2)	
	4–5	With CKD	–	284 (0.6)	–
		No CKD		1244 (0.3)	
	>5	With CKD	–	118 (0.2)	–
		No CKD		599 (0.1)	
Length of stay (LOS) means (25% - 75%)		With CKD	10 (2–7)	23 (7–29)	9 (2–9)
		No CKD	10 (2–11)	20 (6–23)	6 (1–6)
Length of stay (LOS) (Median)		With CKD	5	13	5
		No CKD	5	11	3
Index hospitalization cost (hospital + physician claims) (SD)		With CKD	6397 (18,051)	18,883 (39,649)	5848 (12,034)
		No CKD	5057 (16,879)	16,362 (44,628)	4883 (12,283)
Post index cost (ambulatory care + physician claims) (SD)		With CKD	559 (13,202)	797 (20,171)	539 (12,159)
		No CKD	348 (11,511)	646 (23,808)	337 (10198)
Readmission cost (SD)		With CKD	3310 (12,934)	6133 (19,910)	3001 (11,890)
		No CKD	2038 (11,317)	5888 (23,555)	1796 (10,010)

All differences were statistically significant *p* < 0.05

### Incremental index hospitalization costs (hospital costs and in-hospital physician claims) in patients with CKD

Unadjusted median index hospitalization costs including hospital costs and physician claims within hospital in patients with ≥1 potentially preventable HAC was $18,883 (SD = 39,649), almost three fold greater than patients with no preventable HAC (Table 1). In fully adjusted analyses median incremental index hospitalization costs was $6169 (95% CI; 6003–6336) (Table 2). Costs increased dramatically in a graded fashion with increasing number of potentially preventable HACs; for example, patients with 4–5 potentially preventable complications were associated with incremental costs of $19,083 (95% CI: 18,498–19,667) (Table 2.)

**Table 2:** Adjusted median Incremental in-hospital cost by cost category associated with potentially preventable HACs

# of preventable HACs	In hospital *(95% CI)*	Physician claims *(95% CI)*	Total *(95% CI)*
≥1	4047 (3918–4176)	765 (738–792)	6169 (6003–6336)
One	3191 (3007–3375)	712 (672–751)	3970 (3779–4162)
2–3	7434 (7183–7684)	1323 (1270–1377)	11,769 (11,482–12,056)
4–5	14,609 (14,162–15,057)	2537 (2441–2632)	19,083 (18,498–19,667)
>5	24,639 (24,102–25,175)	5899 (5784–6014)	39,584 (38,675–40,493)

- Adjusted for age, admission type (elective vs urgent), gender, LOS, severity of CKD, non-preventable complications, and16 comorbid conditions

- Reference; admissions without potentially preventable HACs. All analyses were statistically significant (*P_value < 0.05*)

### Incremental healthcare costs within 90 days after discharge (including; physician claims, ambulatory care costs, readmission) in patients with CKD

Unadjusted median ambulatory healthcare costs and physician claims within 90 days after hospital discharge in patients with ≥1 potentially preventable HAC was 50% greater than those patients without potentially preventable complications (Table 1). In patients with ≥1 potentially preventable HACs who were readmitted within 90 days after discharge, the costs of hospital readmission were $6133, two-fold higher than for patients without complications (Table 1). In fully adjusted models, incremental ambulatory care cost and readmission costs associated with ≥1 potentially preventable HAC were $119 (95% CI; 74–164), and $1429 (95% CI; 1150–1709), respectively. Considering all cost categories within 90 days after discharge in patients with ≥1 potentially preventable HAC, adjusted median incremental cost was $1471(95% CI; 844–2099) (Table 3). In fully adjusted analyses, incremental physician claims in patients with potentially preventable HACs was $71 (95% CI; 54–89). (Table 3). Results were similar when readmissions extending beyond the 90 day observation period (*n* = 133) were excluded.

**Table 3:** Adjusted median Incremental cost within 90 days after discharge associated with potentially preventable HACs

Discharge to 90 days cost	Incremental cost (95% CI)
Ambulatory care costs with ≥1 preventable complications	119 (74–164)
Physician claim costs with ≥1 preventable complications	71 (54–89)
Readmission	1429 (1150–1709)
Total	1471 (844–2099)

- Adjusted for age, admission type (elective vs urgent), gender, LOS, severity of CKD, non-preventable complications, and 16 comorbid conditions

- Reference; admissions without potentially preventable HACs. All analyses were statistically significant (*P_value < 0.05*)

### Incremental costs of potentially preventable HACs within 90 days from hospital admission

Within 90 days from hospital admission in CKD patients with ≥1 potentially preventable HACs, unadjusted median healthcare cost was $24,137 (SD = 32,500) compared to $8528 (SD = 18,276) in those patients without preventable HACs. In fully adjusted models median incremental cost in patients with ≥1 potentially preventable HACs was $7522 (95% CI; 7219–7824). Incremental costs increase with the number of complications in a graded fashion, for example in patients with 4 or 5 potentially preventable HACs median incremental cost was $21,882 (95% CI; 20,809–22,955) (Tables 4 and 6). When readmissions extending beyond 90 days (*n* = 198) were excluded similar results were obtained.

**Table 4:** Adjusted median incremental costs in CKD patients with hospital complications within 90 days from hospital admission

		Potentially preventable complications^a^	All complications^b^
Incremental cost (95% CI)*	≥1 complication	7522 (7219–7824)	6612 (6278–6946)
	One	4676 (4332–5020)	4755 (4428–5122)
	2–3	14,184 (13,658–14,73)	12,163 (11,659–12,557)
	4–5	21,882 (20,809–22,955)	21,062 (20,184–21,940)
	>5	38,632 (36,870–40,394)	35,843 (34,733–36,953)

^a^Adjusted for age, admission type (elective vs urgent), gender, LOS, severity of CKD, non-preventable complications, and 16 comorbid conditions

^b^Adjusted for age, admission type (elective vs urgent), gender, severity of CKD, and 16 comorbid conditions

- Reference; admissions without HAC or P-HAC. *All analyses were statistically significant (*P_value < 0.05*)

### Sensitivity analyses

In additional regression analyses, the higher incremental cost of the index hospitalization persisted in all LOS quantiles; in the 0–25% (LOS ≤ 7) percentile, the incremental cost with ≥1 potentially preventable HACs was $3304 (95% CI: 3096–3512) and increased in the subsequent quantiles. (Table 5).

**Table 5:** Median unadjusted and adjusted incremental index hospitalization costs by LOS quantiles in CKD cohort with preventable HACs

Quantile		0–25%	26–50%	51–75%	76–100%
LOS		<7	7 to <13	13 to <29	>29
Median unadjusted costs	*With HAC*	9687	16,576	22,352	48,620
	*No HAC*	4635	8588	11,946	29,127
	*Cost difference*	5052	7988	10,220	19,493
Adjusted incremental costs (95% CI)*		3304 (3096–3512)	5998 (5722–6720)	8775 (8177–9372)	13,749 (12,425–15,073)

- Adjusted for age, admission type (elective vs urgent), gender, LOS, non-preventable complications (where appropriate), and 16 comorbid conditions

- Reference; admissions without potentially preventable HACs. *All analyses were statistically significant (*P_value < 0.05*)

In other sensitivity analyses, generalized linear models with logarithmic transformation and gamma distribution was conducted to examine in-hospital costs; results were similar to the OLS model.

### Incremental costs of potentially preventable HACs within 90 day from hospital admission in cohort without CKD

Further analyses were done to determine the impact of the presence or absence of the CKD in patients with 1 ≥ potentially preventable HACs. A new cohort that included patients without CKD was assembled. In fully adjusted analyses the median incremental costs within 90 days period in patients without CKD who had potentially preventable HACs was $6688 (95% CI; 6612–6723) (Table 6).

**Table 6:** Adjusted median incremental costs within 90 days after hospital admission associated with potentially preventable HACS in patients with and without CKD

	Patients	
	Without CKD	With CKD
Incremental costs of preventable HACs (95% CI) *	6688 (6612–6723)	7522 (7219–7824)

- Adjusted for age, admission type (elective vs urgent), gender, LOS, non-preventable complications, and 16 comorbid conditions

- Reference; admissions without potentially preventable HACs. * Significant differences (*P_value < 0.05*)

## Discussion

We found that patients with potentially preventable complications had substantially greater costs during their index hospitalization as well as in the post-discharge period, and these costs were even greater when potentially preventable complications occurred in patients with CKD. The association of potentially preventable HACs with healthcare costs increased in a graded fashion with increasing number of complications. In patients with CKD, the magnitude of this association was large and the incremental costs of the index hospitalization with 2–3 potentially preventable HACs was a three-fold increase of index hospitalization costs of those patients without potentially preventable complications. As an example of the potential real world implications, while there are acknowledged limitations and potential biases, using the population attributable risk percent we extrapolated our findings to all of North America; we estimate that in 2013 approximately 3.25 million patients with CKD (8.5%) were admitted in North America [19, 20]. If the association of potentially preventable complications and incremental healthcare costs are causal, potentially preventable HACs may be responsible for approximately CA$2.4 billion in additional costs per year with the relative value of CA$2.7 billion in 2016. If prevention leads to averting even a fraction of attributable costs this would represent considerable savings, in addition to the potential for better patient outcomes.

To our knowledge, no study has determined the incremental costs of potentially preventable HAC in patients with CKD, although other work has examined general hospitalized patient populations. However, our findings, including the magnitude of incremental cost in patients with ≥1 potentially preventable HACs in a cohort of patients with and without CKD, are congruent with these other studies in general inpatient populations. In a retrospective study conducted in Alberta, Canada in 2008, 24% of hospitalization episodes had at least one HAC, and was associated with additional costs of C$10,866, more than double the mean cost of an uncomplicated admission [4]. Our incremental results are slightly lower, but we have reported median incremental cost (numerically lower than the mean in skewed data). Internationally, other studies also report the economic impact of HAC. Using the definition of potentially preventable HACs developed by 3 M Health Information Systems, 6% of Medicaid adult and obstetric populations had at least one potentially preventable HAC in fiscal year 2012. It was estimated that the economic burden of potentially preventable HACs was $97.4 million, or 3.7% of total hospital costs of caring for these patients [8].

Targeted strategies to prevent HACs may be effective in some settings [21–23], and could lead to a corresponding decrease in healthcare costs. In a general hospitalized population various strategies have been implemented, including environmental efforts to control hospital infections and procedures for management of patients with foley catheters, leading to a relatively high preventable HAC rate (48.73 and 58.17 per 1000 discharge) reduced to 32.36 and 48.15, respectively [22]. Another study determined the impact of payment reform by the US Centers for Medicare and Medicaid (CMS) where no payment to hospitals would be made for selected preventable complications. Hospitals implemented preventive strategies in response to these incentives and the rate of some complications decreased including central line associated blood stream infections and catheter associated urinary tract infections [24]. An observational retrospective study by the Agency for Healthcare Research and Quality suggests that, through implementation of specific recommendations on best practices to prevent hospital acquired complications in the US (including prevention of pressure ulcers, catheter induced blood stream infections, deep vein thrombosis, etc.), hospitalized patients had 17% (1.3 million) fewer HACs over a 3-year period 25. This reduction in the rate of potentially preventable HACs was estimated to lead to approximately $12 billion savings in healthcare costs.

Effective strategies to prevent HACs targeted at vulnerable patient populations may result in a proportionately greater reduction of health expenditure. Patients with CKD are at increased risk of complications during hospitalization [10], potentially due to known factors such as impaired coagulation, susceptibility to infection, altered renal handling of medications requiring drug dosing changes and predisposition to drug toxicity, among others. While CKD patients may benefit from implementing general preventive strategies, strategies targeting this readily identifiable high-risk population may lead to greater reduction of potentially preventable HACs more efficiently, and subsequently improve patient and healthcare system outcomes in hospitalized patients.

Strengths of our study include the consideration of all HACs and those deemed to be potentially preventable as exposure variables in a population based cohort of patients with CKD. Prior studies have analyzed only cost within index hospitalization associated with potentially preventable HACs which may underestimate the incremental costs estimation associated with those conditions in short term. Shortly after discharge, services such as physician office visits, emergency department visits, ambulatory care, and readmission may occur as extended consequences of potentially preventable HACs. We adjusted our model for LOS, age, gender, admission type (urgent vs elective), severity of CKD, and comorbid conditions, as well as for hospital complications that are not preventable, the latter of which has not been performed in other studies to the best of our knowledge.

In addition to previous limitations [10], it is also possible that the aggregated costing approach used by Canadian Institute for Health Information may underestimate the incremental cost association with potentially preventable HACs, as their methodology may not fully capture the incremental cost attributable to those complications within a CMG. Secondly, administrative data lacks information regarding unmeasured confounders (such as frailty, blood pressure, etc.), however we captured all important co-morbid conditions that were available. Thirdly, there are limitations and potential biases in using the population attributable risk percent which may alter results of PAR%. Fourthly, administrative data may not be sensitive for some types of hospital acquired conditions. As such, the number of hospital acquired complications is likely to be underestimated, however we do not believe this would invalidate results as incomplete ascertainment would be expected to occur equally in both CKD and non-CKD patients. Other unobserved risk differences may exist between groups of patients examined, and associations observed may be due in part to these unobserved factors. Finally, association of incremental cost and potentially preventable HACs may be mediated by longer LOS which is closely correlated with cost, a potential endogeneity bias. Endogeneity bias may result in inflated estimates of the cost impact of potentially preventable HACs, although our analyses adjusted for available data on days in hospital. Results of regression analyses indicate that conclusions are not altered when analyses are performed by quantiles of LOS where HAC independently leads to incremental cost among patients categorized by LOS. Patients with CKD who had preventable HACs also had more non-preventable complications, compared to those subjects with no preventable HACs. It is not clear why complications cluster in certain patients, but we speculate that this may happen in patients with greater clinical complexity, and complications may accrue as patients stay in hospital longer, or be a follow-on effect of the initial preventable complications. The effect of any preventive strategy on this clustering of complications is unknown. We acknowledge that there is insufficient data to make a definitive determination of what is the ‘correct’ approach (whether non-preventable complications should be a covariate). Our analyses control for these complications, and as If any bias exists it is likely to underestimate this association.

## Conclusion

The presence of ≥1 potentially preventable HACs was associated with incremental healthcare costs. This cost is numerically greater in patients with CKD. Further studies are proposed to examine the effect of evidence-based strategies on the risk of potentially preventable hospital acquired complications, with the goal of improving quality of care and reducing costs.

## Appendix A

### PPC Description

01 Stroke & Intracranial Hemorrhage.

02 Extreme CNS Complications.

03 Acute Pulmonary Edema and Respiratory Failure without Ventilation.

04 Acute Pulmonary Edema and Respiratory Failure with Ventilation.

05 Pneumonia & Other Lung Infections.

06 Aspiration Pneumonia.

07 Pulmonary Embolism.

08 Other Pulmonary Complications.

09 Shock.

10 Congestive Heart Failure.

11 Acute Myocardial Infarction.

12 Cardiac Arrythmias & Conduction Disturbances.

13 Other Cardiac Complications.

14 Ventricular Fibrillation/Cardiac Arrest.

15 Peripheral Vascular Complications Except Venous Thrombosis.

16 Venous Thrombosis.

17 Major Gastrointestinal Complications without Transfusion or Significant Bleeding.

18 Major Gastrointestinal Complications with Transfusion or Significant Bleeding.

19 Major Liver Complications.

20 Other Gastrointestinal Complications without Transfusion or Significant Bleeding.

21 Clostridium Difficile Colitis.

22 Urinary Tract Infection.

23 GU Complications Except UTI.

24 Renal Failure without Dialysis.

25 Renal Failure with Dialysis.

26 Diabetic Ketoacidosis & Coma.

27 Post-Hemorrhagic & Other Acute Anemia with Transfusion.

28 In-Hospital Trauma and Fractures.

29 Poisonings Except from Anesthesia.

30 Poisonings due to Anesthesia.

31 Decubitus Ulcer.

32 Transfusion Incompatibility Reaction.

33 Cellulitis.

34 Moderate Infections.

35 Septicemia & Severe Infections.

36 Acute Mental Health Changes.

37 Post-Operative Infection & Deep Wound Disruption without Procedure.

38 Post-Operative Wound Infection & Deep Wound Disruption with Procedure.

39 Reopening Surgical Site 40 Post-Operative Hemorrhage & Hematoma without Hemorrhage Control Procedure or I&D Procedure 41 Post-Operative Hemorrhage & Hematoma with Hemorrhage Control Procedure or I&D Procedure 42 Accidental Puncture/Laceration During Invasive Procedure 43 Accidental Cut or Hemorrhage During Other Medical Care 44 Other Surgical Complication - Moderate 45 Post-procedure Foreign Bodies.

46 Post-Operative Substance Reaction & Non-O.R. Procedure for Foreign Body.

47 Encephalopathy.

48 Other Complications of Medical Care.

49 Iatrogenic Pneumothrax.

50 Mechanical Complication of Device, Implant & Graft.

51 Gastrointestinal Ostomy Complications.

52 Inflammation & Other Complications of Devices, Implants or Grafts Except Vascular Infection.

53 Infection, Inflammation and Clotting Complications of Peripheral Vascular Catheters and Infusions.

54 Infections due to Central Venous Catheters.

55 Obstetrical Hemorrhage without Transfusion.

56 Obstetrical Hemorrhage with Transfusion.

57 Obstetric Lacerations & Other Trauma without Instrumentation.

58 Obstetric Lacerations & Other Trauma with Instrumentation.

59 Medical & Anesthesia Obstetric Complications.

60 Major Puerperal Infection and Other Major Obstetric Complications.

61 Other Complications of Obstetrical Surgical & Perineal Wounds.

62 Delivery with Placental Complications.

63 Post-Operative Respiratory Failure with Tracheostomy.

64 Other In-Hospital Adverse Events.

## Appendix B

### CIHI CMG+ methodology

Canadian Institute for Health Information (CIHI) in April 2007 introduced the case mix group (CMG+) methodology, which identifies clinically similar and/or homogenous groups of patients based on health care resources used. The most responsible diagnoses or principle condition are used to determine major clinical category (MCCs). In all there are 558 CMG codes and 1588 codes when considering inbuilt complexity adjustment. Applying age groups to those codes the result is 4760 new codes.

In CMG+ methodology, variables of flagged intervention, intervention event, and out- of- hospital intervention were added to base system (CMG) with variables of age category, and comorbidity level. The variables used in determining the complexity levels are: major clinical categories/case mix groups, pre-admission comorbid conditions (type 1 diagnosis), post-admission comorbid conditions (type 2 diagnosis), service transfer diagnosis (type W, X, or Y diagnosis), comorbidity grades, number of body systems involved, number of “complex” comorbidities. Levels of complexity are; 1 – no complexity (0% to 24% impact on resource consumption), 2 – complexity related to chronic diseases (25% to 49% impact on resource consumption), 3 – complexity related to important clinical conditions (75% to 124% impact on resource consumption), 4 – complexity related to life-threatening conditions (125% + impact on resource consumption), 9 – complexity not applied (already captured within the CMG). CIHI methodology applies an age overlay to each CMG: 1) 0 to 17 years old, 2) 18 to 69 years old, 3) 70 plus years old.

Annually CIHI publishes the relative cost of patients types called “Resource Intensity Weights” (RIWs). RIW presents a modelled estimate of the relative resources used by a patient. In CMG+ methodology, RIWs are adjusted for five factors known to have an impact on hospital costs. On average, older patients with more clinical complexity who tend to consume more resources would have greater RIW than patients with fewer health issues. CIHI annually calculates and updates based on data from the discharge abstract database (DAD) and from case cost data provided by some provinces. RIW values in DAD represent relative resource use of different hospitalized patients. (for example: RIW = 10.68, uses 10.68 times more resources than the average case).

Average financial cost a hospital incurs to treat a single inpatient is presented by the Cost Per Weighted Case (CPWC) measure by dividing total inpatients cost for a facility by the total weighted cases in that facility.

### CPWC = Total Inpatient Costs/Total Weighted Cases

The CPWC is calculated and updated annually from CIHI’s Canadian MIS database, based on data provided by all hospitals.

The per patient index hospitalization costs are generated by multiplying CPWC by the average resource intensity weight (RIW) in selected cases for each fiscal year.

Average or typical costs are estimated using average length of stay (ALOS) in hospital. The trim point is the point after which a patient’s length of stay (LOS) is considered to be an outlier. Long-stay cases are defined as those that stay longer than the nationally-determined trim point, which is determined separately for each CMG using the following formula: Trim Point = Q3 + Multiplier × (Q3 − Q1), where:

• Q3 is the third quartile length of stay for the CMG (that is, where 75% of patients have a shorter stay);

• Q1 is the first quartile length of stay for the CMG; and

• The multiplier is a number that is set each year so that a specific proportion (4.5%) of cases overall (not by CMG) are defined as long stay.

The cost of those admissions with LOS more than ALOS, is estimated by CIHI as a per diem amount based on episodes with similar CMG and factor assignments allocated to each day beyond the trim point.

CIHI has also developed the National Ambulatory Care Reporting System (NACRS) which includes data for all hospital-based and community-based ambulatory care including: day surgery, emergency departments, outpatient and community-based clinics. Specific categories include emergency visits, ambulatory interventions, rehabilitation and clinic visits with the exception of telephone visits and direct diagnostic imaging. Emergency and ambulatory care account for the largest-volume patient activity, making these services a main component of the continuum of health services in Canada.

Hospitals report ambulatory data in varying levels of detail enabling CIHI or individual provinces to develop RIWs for an outpatient classification system called the Comprehensive Ambulatory Care Classification System (CACS). Only a small number of hospitals in Ontario, Alberta, and British Columbia collect outpatient costs — that is, costs associated with NACRS records. Collected data elements in the NACRS database includes patient identifiers, mode of care (emergency department, surgery, clinics, diagnostic imaging), diagnoses, interventions, time elements (time of arrival, time seen by clinician), and disposition of patient (discharged, admitted). Cost data, which is collected by only a few hospitals, includes direct costs (nursing, diagnostic tests, operating, and recovery room), functional centre indirect costs (meals, facilities management, plant operation), and costs for patient-specific drugs and supplies. CIHI estimates a series of resource weights for each of these CACS groups that have been developed in tandem with in-patient RIWs.

## Abbreviations

AKI: Acute kidney injury
CIHI: Canadian Institute for Health Information
CKD: Chronic kidney disease
CMG: Case mix group
COPD: Chronic obstructive pulmonary disease
ESRD: End stage renal disease
HAC: Hospital acquired complications
ICD 10 CA: International classification of Diseases 10 Canadian modification
ICD 9 CM: International classification of Diseases 9 Clinical modification
LOS: Length of stay
OLS: Ordinary Least Squares
